# Deep Personality Trait Recognition: A Survey

**DOI:** 10.3389/fpsyg.2022.839619

**Published:** 2022-05-06

**Authors:** Xiaoming Zhao, Zhiwei Tang, Shiqing Zhang

**Affiliations:** ^1^Institute of Intelligence Information Processing, Taizhou University, Taizhou, Zhejiang, China; ^2^School of Faculty of Mechanical Engineering and Automation, Zhejiang Sci-Tech University, Hangzhou, China

**Keywords:** personality trait recognition, personality computing, deep learning, multimodal, survey

## Abstract

Automatic personality trait recognition has attracted increasing interest in psychology, neuropsychology, and computer science, etc. Motivated by the great success of deep learning methods in various tasks, a variety of deep neural networks have increasingly been employed to learn high-level feature representations for automatic personality trait recognition. This paper systematically presents a comprehensive survey on existing personality trait recognition methods from a computational perspective. Initially, we provide available personality trait data sets in the literature. Then, we review the principles and recent advances of typical deep learning techniques, including deep belief networks (DBNs), convolutional neural networks (CNNs), and recurrent neural networks (RNNs). Next, we describe the details of state-of-the-art personality trait recognition methods with specific focus on hand-crafted and deep learning-based feature extraction. These methods are analyzed and summarized in both single modality and multiple modalities, such as audio, visual, text, and physiological signals. Finally, we analyze the challenges and opportunities in this field and point out its future directions.

## Introduction

In (Vinciarelli and Mohammadi, 2014), the concept of personality can be defined as “*personality is a psychological construct aimed at explaining the wide variety of human behaviors in terms of a few, stable and measurable individual characteristics*.” In this case, personality can be characterized as a series of traits. The trait theory (Costa and McCrae, 1998) aims to predict relatively stable measurable aspects in the people’s daily lives on the basis of traits. It is used to measure human personality traits, that is, customary patterns of human behaviors, ideas, and emotions which are relatively kept steady over time. Some previous works explored the interaction between personality and computing by means of measuring the connection between traits and the used techniques (Guadagno et al., 2008; Qiu et al., 2012; Quercia et al., 2012; Liu et al., 2016; Kim and Song, 2018; Masuyama et al., 2018; Goreis and Voracek, 2019; Li et al., 2020a). The central idea behind these works is that users aim to externalize their personality by the way of using techniques. Accordingly, personality traits can be identified as predictive for users’ behaviors.

At present, various personality trait theories have been developed to categorize, interpret and understand human personality. The representative personality trait theories contain the Cattell Sixteen Personality Factor (16PF; Cattell and Mead, 2008), the Hans Eysenck’s psychoticism, extraversion and neuroticism (PEN; Eysenck, 2012), Myers–Briggs Type Indicator (MBTI; Furnham and Differences, 1996), Big-Five (McCrae and John, 1992), and so on. So far, the widely used measure for automatic personality trait recognition is the Big-Five personality traits. The Big-Five (McCrae and John, 1992) model measures personality through five dipolar scales:

“Extraversion”: outgoing, energetic, talkative, active, assertive, etc.

“Neuroticism”: worrying, self-pitying, unstable, tense, anxious, etc.

“Agreeableness”: sympathetic, forgiving, generous, kind, appreciative, etc.

“Conscientiousness”: responsible, organized, reliable, efficient, planful, etc.

“Openness”: artistic, curious, imaginative, insightful, original, wide interests, etc.

In recent years, personality computing (Vinciarelli and Mohammadi, 2014) has become a very active research subject that focuses on computational techniques related to human personality. It mainly addresses three fundamental problems: automatic personality trait recognition, perception, and synthesis. The first one aims at correctly identifying or predicting the actual (self-assessed) personality traits of human beings. This allows the construction of an apparent personality (or first impression) of an unacquainted individual. Automatic personality trait perception concentrates on analyzing the different subjective factors that affect the personality perception for a given individual. Automatic personality trait synthesis tries to realize the generation of artificial personalities through artificial agents and robots. This paper focuses on the first problem of personality computing, that is, automatic personality trait recognition, due to its potential applications to emotional and empathetic virtual agents in human–computer interaction (HCI).

Most prior works focus on personality trait modeling and prediction from different cues, both behavioral and verbal. Therefore, automatic personality trait recognition takes into account multiple input modalities, such as audio, text, and visual cues. In 2015, the INTERSPEECH Speaker Trait Challenge (Schuller et al., 2015) provided a unified test run for predicting the Big-Five personality traits, likability, and pathology of speakers, and meanwhile presented a performance comparison of computational models with the given data sets, and extracted features. In 2016, the well-known European Conference on Computer Vision (ECCV) released a benchmark open-domain personality data set, that is, Cha-Learn-2016, to organize a competition of personality recognition (Ponce-López et al., 2016).

Automatic personality trait recognition from social media contents has recently become a challenging issue and attracted much attention in the fields of artificial intelligence and computer vision, etc. So far, several surveys on personality trait recognition have been published in recent years. Specially, Vinciarelli and Mohammadi (2014) provided the first review on personality computing, related to automatic personality trait recognition, perception, and synthesis. This review was organized from a more general point of view (personality computing). Junior et al. (2019), also presented a survey on vision-based personality trait analysis from visual data. This survey focused on the single visual modality. Moreover, these two surveys concentrate on classical methods, and recently emerged deep learning techniques (Hinton et al., 2006) have seldom been reviewed. Very recently, Mehta et al. (2020b) presented a brief review deep learning-based personality trait detection. Nevertheless, they did not provide a summary on personality trait databases and technical details on deep learning techniques. Therefore, this paper gives a comprehensive review for personality trait recognition from a computational perspective. In particular, we focus on reviewing the recent advances of existing both single and multimodal personality trait recognition methods between 2012 and 2022 with specific emphasis on hand-crafted and deep learning-based feature extraction. We aim at providing a newcomer to this field, a summary of the systematic framework, and main skills for deep personality trait recognition. We also examine state-of-the-art methods that have not been mentioned in prior surveys.

In this survey, we have searched the published literature between January 2012, and February 2022 through Scholar.google, ScienceDirect, IEEEXplore, ACM, Springer, PubMed, and Web of Science, on the basis of the following keywords: “personality trait recognition,” “personality computing,” “deep learning,” “deep belief networks,” “convolutional neural networks,” “recurrent neural networks,” “long short-term memory,” “audio,” “visual,” “text,” “physiological signals,” “bimodal,” “trimodal,” and “multimodal.” There is no any language restriction for the searching process. We designed and conducted this systematic survey by complying with the PRISMA statement (Sarkis-Onofre et al., 2021) in an effort to improve the reporting of systematic reviews. Eligibility criteria of this survey contain the suitable depictions of different hand-crafted and deep learning-based feature extraction methods for personality trait recognition in both single modality and multiple modalities.

**Figure 1:**
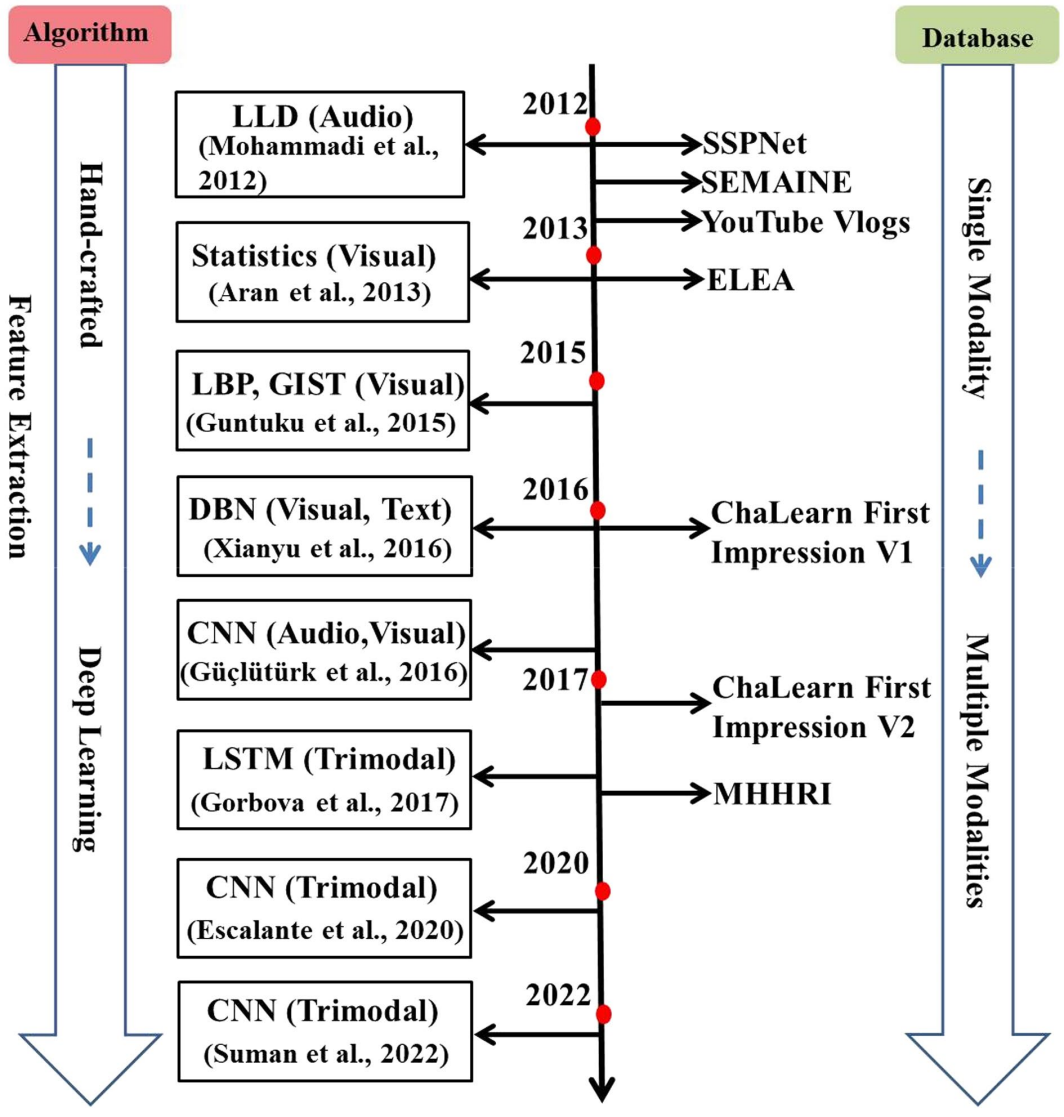
The evolution of personality trait recognition with feature extraction algorithms and databases. From 2012 to 2022, feature extraction algorithms have changed from hand-crafted to deep learning. Meanwhile, the developed databases have evolved from single modality (audio or visual) to multiple modalities (audio, visual, text, etc.).

It is noted that a basic personality trait recognition system generally consists of two key parts: feature extraction and personality trait classification or prediction. Feature extraction can be divided into hand-crafted and deep learning-based methods. For personality trait classification or prediction, the common classifiers/regressors, such as Support Vector Machines (SVM) and linear regressors, are usually used. In this survey, we focus on the advances of feature extraction algorithms ranging from 2012 to 2022 in a basic personality trait recognition system. Figure 1 shows the evolution of personality trait recognition with feature extraction algorithms and databases.

In this work, our contributions can be summarized as follows:

1. We provide an up-to-date literature survey on deep personality trait analysis from a perspective of both single modality and multiple modalities. In particular, this work focuses on a systematical single and multimodal analysis of human personality. To the best of our knowledge, this is the first attempt to present a comprehensive review covering both single and multimodal personality trait analysis related to hand-crafted and deep learning-based feature extraction algorithms in this field.
2. We summarize existing personality trait data sets and review the typical deep learning techniques and its recent variants. We present the significant advances in single modality personality trait recognition related to audio, visual, text, etc., and multimodal personality trait recognition related to bimodal and trimodal modalities.
3. We analyze and discuss the challenges and opportunities faced to personality trait recognition and point out future directions in this field.

The remainder of this paper is organized as follows. Section “Personality Trait Databases” describes the available personality trait data sets. Several typical deep learning techniques and its recent variants are reviewed in detail in Section “Review of Deep Learning Techniques.” Section “Review of Single Modality Personality Trait Recognition Techniques” introduces the related techniques of single modality personality trait recognition. Section “Multimodal Fusion for Personality Trait Recognition” provides the details of multimodal fusion for personality trait recognition. Section “Challenges and Opportunities” discusses the challenges and opportunities in this field. Finally, the conclusions are given in Section “Conclusion.”

## Personality Trait Databases

To evaluate the performance of different methods, a variety of personality trait data sets, as shown in Table 1, are collected for automatic personality trait recognition. These representative data sets are described as follows.

**Table 1:** Comparisons of representative personality trait recognition databases.

Data set	Year	Brief description	Central issues	Labels	Modality	Environment
SSPNet (Mohammadi and Vinciarelli, 2012)	2012	640 audio clips from 322 speakers	Personality trait assessment from speech	BFI-10 personality assessment questionnaire, Big-Five impressions	Audio	Uncontrolled
SEMAINE (McKeown et al., 2012)	2012	959 conversations from 150 participants	Face-to-face conversations with sensitive artificial listener agents	Five affective dimensions and 27 associated categories	Audio-visual	Controlled
YouTube Vlogs (Biel and Gatica-Perez, 2012)	2012	2,269 videos from 469 different vloggers	Conversational vlogs and apparent personality trait analysis	Big-Five impressions	Audio-visual	Uncontrolled
ELEA (Sanchez-Cortes et al., 2013)	2013	40 meeting sessions with about 10 h of recordings (148 participants)	Small group interactions and emergent leadership	Big-Five impressions	Audio-visual	Controlled
ChaLearn First Impression V1 (Ponce-López et al., 2016)	2016	10,000 videos from 2,762 YouTube users	Apparent personality trait analysis	Big-Five impressions	Audio-visual	Uncontrolled
ChaLearn First Impression V2 (Escalante et al., 2017)	2017	An extended version of [5], including the newly added hirability impressions and audio transcripts	Apparent personality trait and hirability impressions	Big-Five impressions, job interview variable, and transcripts	Multimodal	Uncontrolled
MHHRI (Celiktutan et al., 2017)	2017	12 interaction sessions (about 4 h) from 18 participants	Personality and engagement during HHI and HCI	Self/acquaintance assessed Big-Five, and engagement	Multimodal	Controlled
UDIVA (Palmero et al., 2021)	2021	188 dyadic sessions (90.5 h) from 147 participants	Context-aware personality inference in dyadic scenarios	Big-Five scores, sociodemographics, mood, fatigue, relationship type	Multimodal	Controlled

### SSPNet

The SSPNet (Mohammadi and Vinciarelli, 2012) speaker personality corpus is the biggest up-to-date data set for the assessment of personality traits in speech signals. It contains 640 audio clips from 322 speakers with a sampling rate of 8 kHz. These audio clips are randomly derived from the French news in Switzerland. Most of them are 10 s long. In addition, 11 judges are invited to annotate every clip by means of filling out the BFI-10 personality evaluation questionnaire (Rammstedt and John, 2007). A score is calculated for every Big-Five personality trait on the basis of the questionnaire. The judges are not familiar with French and thus could not be affected by linguistic cues.

### Emergent Leader

The Emergent LEAder (ELEA; Sanchez-Cortes et al., 2013) data set comprises of 40 meeting sessions associated with about 10 h of recordings. It consists of 28 four-person conferences as well as 12 three-person conferences in newly constructed groups, in which previously unacquainted persons are included. The mean age for 148 participants (48 women and 100 men) is 25.4 years old. All the participants at the ELEA conferences are required to take part in a winter survival task, but are not assigned any special role. Audio recordings are collected by using a microphone, and the audio sampling rate is 16 kHz. Video recordings are gathered with two setup settings: a static setting with six cameras, and a portable setting with two webcams. The video frame rates for these two settings are separately 25 fps and 30 fps, respectively.

### SEMAINE

The SEMAINE (McKeown et al., 2012) audio-visual data set contains 150 participants (57 men and 93 women) with a mean age of 32.8 years old. These participants are undergraduate and postgraduate students from eight different nations. The representative conversation duration for Solid SAL and Semi-automatic SAL is approximately 30 min. A total of 959 conversations with individual SAL characters are collected, each of which lasts about 5 min, although there are large individual differences. The Automatic SAL conversation lasts almost 1 h with eight-character interaction per 3 min. Participants interacted with both versions of the system and finished psychometric measures at an interval of 10–15 min.

### YouTube Vlogs

The YouTube Vlogs (Biel and Gatica-Perez, 2012) data set comprises of 2,269 videos with a total of 150 h. These videos, ranging from 1 to 6 min in length, come from 469 different vloggers. It contains video metadata and viewer comments gathered in 2009 (Biel and Gatica-Perez, 2010). The video samples are collected with keywords like “vlogs” and “vlogging.” Meanwhile, the recording setting is that a participant is talking to a camera displaying the participant’s head and shoulder. The recording contents contain various topics, such as personal video blogs, film, product comments, and so on.

### ChaLearn First Impression V1-V2

The ChaLearn First Impression data set has been developed into two versions: the ChaLearn First Impression V1 (Ponce-López et al., 2016), and the ChaLearn First Impression V2 (Escalante et al., 2017): The ChaLearn First Impression V1 contains 10,000 short video clips, collected from about 2,762 YouTube high-definition videos of persons who are facing and speaking to the camera in English. Each video has a resolution of 1,280 × 720, and a length of 15 s. The involved persons have different genders, ages, nationalities, and races. In this case, the task of predicting apparent personality traits becomes more difficult and challenging. The ChaLearn First Impression V2 (Escalante et al., 2017) is an extension of the ChaLearn First Impression V1 (Ponce-López et al., 2016). In this data set, the new variable of “job interview” is added for prediction. The manual transcriptions associated with the corresponding audio in videos are also provided.

### Multimodal Human–Human–Robot Interactions

The multimodal human–human–robot interactions (MHHRI; Celiktutan et al., 2017) data set contains 18 participants (nine men and nine women), most of whom are graduate students and researchers. It includes 12 interaction conversations (about 4 h). Each interactive conversation has 10–15 min and is recorded with several sensors. For recording first-person videos, two liquid image egocentric cameras are located on the participants’ forehead. For RGB-D recordings, two static Kinect depth sensors are placed opposite to each other for capturing the entire scene. For audio recordings, the microphone in the egocentric cameras is used. Additionally, participants are required to wear a Q-sensor with Affectiva for recording physiological signals, such as electrodermal activity (EDA).

### Understanding Dyadic Interactions From Video and Audio Signals

The understanding dyadic interactions from video and audio signals (UDIVA; Palmero et al., 2021) data set, comprises of 90.5 h of non-scripted face-to-face dyadic interactions between 147 participants (81 men and 66 women) from 4 to 84 years old. Participants were distributed into 188 dyadic sessions. This data set was recorded by using multiple audio-visual and physiological sensors. The raw audio frame rate is 44.1 kHz. Video recordings are collected from 6 HD tripod-mounted cameras with a resolution of 1,280 × 720. They adopted questionnaire based assessments, including sociodemographic, self- and peer-reported personality, internal state, and relationship profiling from participants.

From Table 1, we can see that the representative personality trait recognition databases are developed from the single modality (audio), bimodality (audio-visual), and multiple modalities. For obtaining the ground-truth scores of personality traits on these databases, personality questionnaires are presented to the users for annotations. Nevertheless, such subjective annotations with personality questionnaires may affect the reliability of trained models on these databases.

## Review of Deep Learning Techniques

In recent years, deep learning techniques have been an active research subject and obtained promising performance in various applications, such as object detection and classification, speech processing, natural language processing, and so on (Yu and Deng, 2010; LeCun et al., 2015; Schmidhuber, 2015; Zhao et al., 2015). In essence, deep learning methods aim to achieve high-level abstract representations by means of hierarchical architectures of multiple non-linear transformations. After implementing feature extraction with deep learning techniques, the Softmax (Sigmoid) function is usually for classification or prediction. In this section, we briefly review several representative deep learning methods and its recent variants, which can be potentially used for personality trait analysis.

### Deep Belief Networks

Deep belief networks (DBNs; Hinton et al., 2006) developed by Hinton et al. in 2006, are a generative model that aim to capture a high-order hierarchical feature representation of input data. The conventional DBN is a multilayered deep architecture, which is built by a sequence of superimposed restricted Boltzmann machines (RBMs; Freund and Haussler, 1994). A RBM is a two-layer generative stochastic neural network consisting of a visual layer and a hidden layer. These two layers in a RBM constitute a bipartite graph without any lateral connection. Training a DBN needs two-stage steps: pretraining and fine-tuning. Pretraining is realized by means of an efficient layer-by-layer greedy learning strategy (Bengio et al., 2007) in an unsupervised manner. During the pretraining process, a contrastive divergence (Hinton, 2002; CD) algorithm is adopted to train RBMs in a DBN to enable the optimization of the weights and bias of DBN models. Then, fine-tuning is performed to update the network parameters by using the back propagation (BP) algorithm.

Several improved versions of DBNs are developed in recent years. Lee et al. (2009), proposed a convolutional deep belief network (CDBN) for full-sized images, in which multiple max-pooling based convolutional RBMs were stacked on the top of one another. Wang et al. (2018) presented a growing DBN with transfer learning (TL-GDBN). TL-GDBN aimed to grow its network structure by means of transferring the learned feature representations from the original structure to the newly developed structure. Then, a partial least squares regression (PLSR)-based fine-tuning was implemented to update the network parameters instead of the traditional BP algorithm.

### Convolutional Neural Networks

Convolutional neural networks (CNNs) were originally proposed by LeCun et al. (1998) in 1998, and initially developed as an advanced version of deep CNNs, such as AlexNet (Krizhevsky et al., 2012) in 2012. The basic structure of CNNs comprises of convolutional layers, pooling layers, as well as fully connected (FC) layers. CNNs usually have multiple convolutional and pooling layers, in which pooling layers are frequently followed by convolutional layers. The convolutional layer adopts a number of learnable filters to perform convolution operation on the whole input image, thereby yielding the corresponding activation feature maps. The pooling layer is employed to reduce the spatial size of produced feature maps by using non-linear down-sampling methods for translation invariance. Two well-known used pooling strategies are average pooling and max-pooling. The FC layer, in which all neurons are fully connected, is often placed at the end of the CNN network. It is used to activate the previous layer for producing the final feature representations and classification.

In recent years, several advanced versions of deep CNNs have been presented in various applications. The representative deep CNN models include AlexNet (Krizhevsky et al., 2012), VGGNet (Simonyan and Zisserman, 2014), GoogleNet (Szegedy et al., 2015), ResNet (He et al., 2016), DenseNet (Huang et al., 2017), and so on. In particular, DenseNet (Huang et al., 2017), in which each layer is connected to each other layer in a feed-forward manner, has been proved that it beats most deep models on objection recognition tasks with less network parameters. Table 2 presents the comparisons of the configurations and characteristics of these typical deep CNNs, as described below.

**Table 2:** Comparisons of deep CNN models and its configurations.

	AlexNet	VGGNet	GoolgeNet	ResNet	DenseNet
Year	2012	2015	2015	2016	2017
layers (Conv. + FC)	5 + 3	19 + 3	21 + 1	151 + 1	264 + 1
Conv. kernel	11,5,3	3	7,1,3,5	7,1,3,5	7,1,3
Dropout	√	√	√	√	√
Inception	×	×	√	×	×
DA	√	√	√	√	√
BN	×	×	×	√	√

Conv., convolution; DA, data augmentation; BN, batch normalization. The number of layers is the used maximum in deep models.

Compared with the above-mentioned deep CNNs processing 2D images, the recently developed 3D-CNNs (Tran et al., 2015) aim to learn temporal-spatio feature representations by using 3D convolution operations on large-scale video data sets. Some improved versions of 3D-CNNs are also recently proposed to reduce the computation complexity of 3D convolutions. Yang et al. (2019) provided an asymmetric 3D-CNN on the basis of the proposed MicroNets, in which a set of local 3D convolutional networks were adopted so as to incorporate multiscale 3D convolution branches. Kumawat and Raman (2019) proposed a LP-3DCNN in which a rectified local phase volume (ReLPV) block was used to replace the conventional 3D convolutional block. Chen et al. (2020) developed a frequency domain compact 3D-CNN model, in which they utilized a set of learned optimal transformation with few network parameters to implement 3D convolution operations by converting the time domain into the frequency domain.

### Recurrent Neural Networks

Recurrent neural networks (RNNs; Elman, 1990) are a single feed-forward neural network for capturing temporal information, and thus suitable to deal with sequence data. RNNs contain recurrent edges connecting adjacent time steps, thereby providing the concept of time in this model. In addition, RNNs share the same network parameters across all time steps. For training RNNs, the traditional back propagation through time (BPTT; Werbos, 1990) was usually adopted.

Long short-term memory (LSTM; Hochreiter and Schmidhuber, 1997), proposed by Hochreiter and Schmidhuber in 1997, is a relatively new recurrent network architecture, which is combined with a suitable gradient-based learning fashion. Specially, LSTMs aim to alleviate the gradient vanishing and exploding problems produced during the procedure of training RNNs. There are three types of gates in a LSTM cell unit: input gate, forget gate, and output gate. Input gate is used to control how much of the current input data is flowing into the memory unit of the network. Forget gate, as a key component of the LSTM cell unit, is used for controlling which information to keep and which to forget, and somehow avoiding the gradient loss and explosion problems. Output gate controls the effect of the memory cell on the current output value. On the basis of these three special gates, LSTMs have an ability of modeling long-term dependencies of sequence data, such as video sequences.

In recent years, a variant of LSTMs called gated recurrent unit (GRU; Chung et al., 2014), was developed by Chung et al. in 2014. GRU makes every recurrent unit to adaptively model long-term dependencies of different time scales. Different from the LSTM unit, GRU does not have a separate memory cell inside the unit. In addition, combining CNNs with LSTMs becomes a research trend. In particular, Zhao et al. (2019) proposed a Bayesian graph based a convolution LSTM for identifying skeleton-based actions. Zhang et al. (2019) developed a multiscale deep convolutional LSTM for speech emotion classification.

## Review of Single Modality Personality Trait Recognition Techniques

Automatic personality trait recognition aims to adopt computer science techniques to realize the modeling of personality trait recognition problems in cognitive science. It is one of the most important research subjects in the field of personality computing (Vinciarelli and Mohammadi, 2014; Junior et al., 2018). According to the types of input data, automatic personality trait recognition can be divided into: single modality and multiple modalities. In particular, it contains the single audio or visual personality trait recognition, and multimodal personality trait recognition, integrating multiple modal behavior data, such as audio, visual, and text information.

### Audio-Based Personality Trait Recognition

Table 3 presents a brief summary of existing literature related to audio-based personality trait recognition.

**Table 3:** A brief summary of audio-based on personality trait recognition.

Year	Authors	Feature descriptions
2012	Mohammadi et al.	Pitch, formants, energy, and speaking rate
2016	An et al.	Interspeech-2013 ComParE feature set
2017	Su et al.	Wavelet-based multiresolution analysis and CNNs for feature extraction
2019	Hayat et al.	Fine-tuning the pretrained AudioSet for feature extraction
2020	Carbonneau et al.	Learning feature dictionary from the extracted patches in speech spectrograms

The early-used audio features for automatic personality trait recognition are hand-crafted low-level descriptive (LLD) features, such as prosody (intensity, pitch), voice quality (formants), spectral features (Mel Frequency Cepstrum Coefficients, MFCCs), and so on. Specially, Mohammadi and Vinciarelli (2012) utilized the LLD features, such as pitch, formants, energy, and speaking rate to detect personality traits in audio clips with less than 10 s. They adopted Logistic Regression to identify whether an audio clip exceeded the average score for each of the Big-five personality traits. In (An et al., 2016), 6,373 acoustic–prosodic features like the Interspeech-2013 ComParE feature set (Schuller et al., 2013) were extracted as an input of the SVM classifier for identifying the Big-Five personality traits. In (Carbonneau et al., 2020), the authors learned a discriminating feature dictionary from the extracted patches in the speech spectrograms, followed by the SVM classifier for the classification of the Big-Five personality traits.

**Figure 2:**
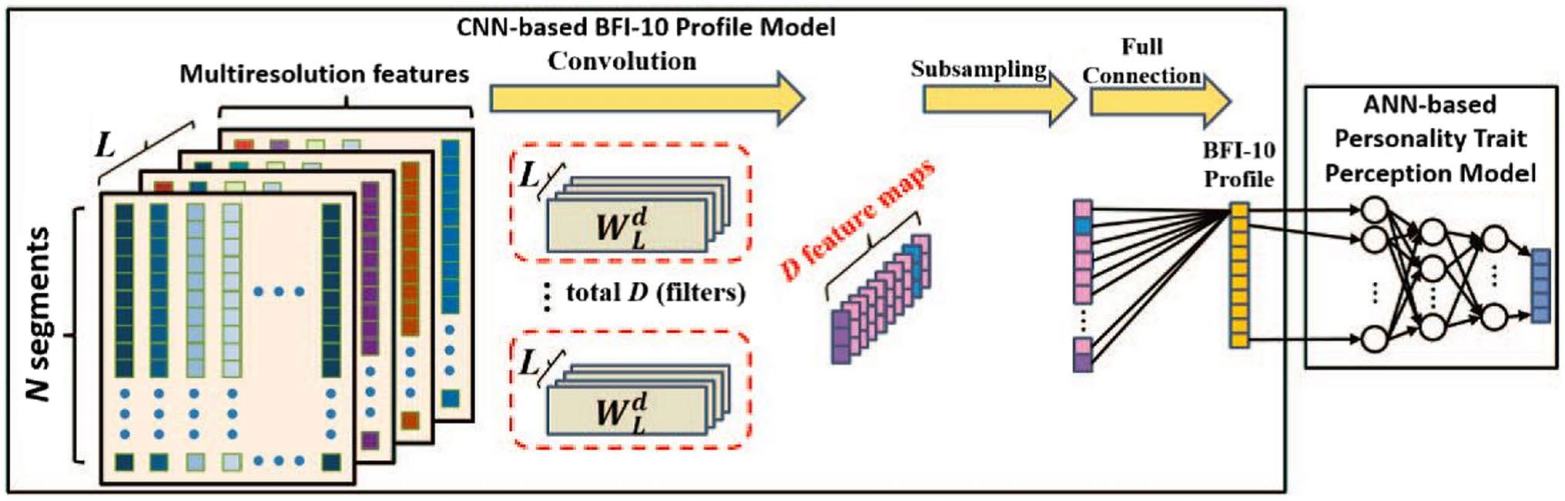
The used CNN scheme for personality trait perception from speech signals (Su et al., 2017).

The recently used audio features for automatic personality trait recognition are deep audio features extracted by deep learning techniques. Su et al. (2017) proposed to employ wavelet-based multiresolution analysis and CNNs for personality trait perception from speech signals. Figure 2 presents the details of the used CNN scheme. The wavelet transform was adopted to decompose the original speech signals at different levels of resolution. Then, based on the extracted prosodic acoustic features, CNNs were leveraged to produce the profiles of the Big-Five Inventory-10 (BFI-10) for a quantitative measure, followed by artificial neural networks (ANNs) for personality trait recognition. Hayat et al. (2019) fine-tuned a pretrained CNN model called AudioSet to learn an audio feature representation for predicting the Big-five personality trait scores of a speaker. They showed the advantages of CNN-based learned features over hand-crafted features.

### Visual-Based Personality Trait Recognition

According to the type of vision-based input data, visual-based personality trait recognition can be categorized into two types: static images and dynamic video sequences. Visual feature extraction is the key step related to the input static images and dynamic video sequences for personality trait recognition. Table 4 provides a brief summary of existing literature related to visual-based (static images, and dynamic video sequences) personality trait recognition.

**Table 4:** A brief summary of visual-based on personality trait recognition.

Visual type	Year	Authors	Feature descriptions
Static images	2015	Guntuku et al.	LBP, GIST, aesthetic features
	2016	Yan et al.	HOG, SIFT, LBP
	2017	Zhang et al.	Fine-tuning the pretrained VGG-face model for facial feature extraction
	2017	Segalin et al.	Fine-tuning the pretrained AlexNet and VGG-16 for aesthetic attributes
	2020	Rodríguez et al.	Trained a ResNet-50 to derive personality representations from the posted images
	2021	Fu et al.	An improved ASM model for facial feature extraction, followed by a DBN
Dynamic video sequences	2012	Biel et al.	Facial activity statistics based on frame-by-frame estimation
	2013	Aran et al.	Statistical information derived from the weighted motion energy images
	2014	Teijeiro-Mosquera, et al.	Four sets of behavioral cues, such as statistic, THR, HMM, and WTA cues
	2016	Gürpinar et al.	Fine-tuning the pretrained VGG-19 to extract deep facial and scene features
	2017	Ventura et al.	An extension of DAN for facial feature extraction in videos
	2019	Beyan et al.	Deep visual activity-based features derived from key-dynamic images in videos

#### Static Images

As far as static image-based personality trait recognition is concerned, researchers have found that a facial image presents most of meaningful descriptive cues for personality trait recognition (Willis and Todorov, 2006). Hence, the extracted visual features involve in the analysis of facial features for personality trait prediction. In (Guntuku et al., 2015), the authors proposed to leverage several low-level features of facial images, such as color histograms, local binary patterns (LBP), global descriptor (GIST), and aesthetic features, to train the SVM classifier for detecting mid-level clues (gender, age). Then, they predicted the Big-five personality traits of users in self-portrait images with the lasso regressor. Yan et al. (2016) investigated the connection between facial appearance and personality impression in the manner of trustworthy. They obtained middle-level cues through clustering methods from different low-level features, such as histogram of oriented gradients (HOG), scale-invariant feature transform (SIFT), LBP, and so on. Then, a SVM classifier was used to exploit the connection between facial appearance and personality impression.

In recent years, CNNs were also widely used for facial feature extraction on static image-based personality trait recognition tasks. Zhang et al. (2017) presented an end-to-end CNN structure *via* fine-tuning a pretrained VGG-face model for feature learning so as to predict personality traits and intelligence jointly. They aimed to explore whether self-reported personality traits and intelligence can be jointly measured from facial images. Segalin et al. (2017) explored the linking the Big-Five personality traits and preferred images in the Flickr social network through image understanding and a deep CNN framework. In particular, they fine-tuned the pretrained AlexNet and VGG-16 modal to capture the aesthetic attributes of the images characterizing the personality traits associated with those images. They changed the last layer of the AlexNet and VGG-16 model to adapt them to a binary classification problem. Experiments results showed that the characterization of each image can be locked within the CNN layers, thereby discovering entangled attributes, such as the aesthetic and semantic information for generalizing the patterns that identify a personality trait. Rodríguez et al. (2020) presented a personality trait analysis in social networks by using a weakly supervised learning method of shared images. They trained a ResNet-50 network to derive personality representations from the posted images in social networks, so as to infer whether the personality scores from the posted images are correlated to those scores obtained from text. For predicting personality traits, the images without manually labeling were used for training the ResNet-50 model. Experiment results indicate that people’s personality is not only related to text, but also with the image content. Fu and Zhang (2021) provided a personality trait recognition method by using active shape model (ASM) localization and DBNs. They employed an improved ASM model to extract facial features, followed by a DBN which was used to train and classify the students’ four personality traits.

#### Dynamic Video Sequences

Dynamic video sequences consist of a series of video image frames, thereby providing temporal information and scene dynamics. This brings about certain useful and complementary cues for personality trait analysis (Junior et al., 2019).

In (Biel et al., 2012), the authors investigated the connection between facial expressions and personality of vloggers in conversation videos (vlogs) from a subset of existing YouTube vlog data set (Biel and Gatica-Perez, 2010). They employed a computer expression recognition toolbox to identify the categories of facial expressions of vloggers. They finally adopted a SVM classifier to predict personality traits in conjunction with facial activity statistics on the basis of frame-by-frame estimation. The results indicate that extraversion has the highest utilization of activity cues. This is consistent with previous findings (Biel et al., 2011; Biel and Gatica-Perez, 2012). Aran and Gatica-Perez (2013) adopted the social media contents from conversational videos for analyzing the specific trait of extraversion. To address this issue, they integrated the ridge regression with a SVM classifier on the basis of statistical information derived from the weighted motion energy images. In (Teijeiro-Mosquera et al., 2014), the relations between facial expressions and personality impressions were investigated as an extended version of the used method (Biel et al., 2012). To characterize face statistics, they derived four sets of behavioral cues, such as statistic-based cues, Threshold (THR) cues, Hidden Markov Models (HMM) cues, and Winner Takes All (WTA) cues. Their research indicates that when multiple facial expression clues are significantly correlated with a certain number of the Big-Five traits, they could only obviously predict the particular trait of extraversion.

**Figure 3:**
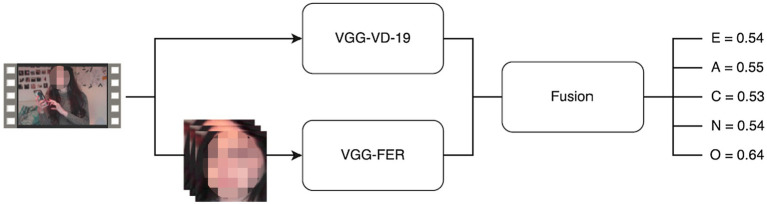
The flowchart of personality trait prediction by using deep facial and scene feature representations (Gürpınar et al., 2016).

**Figure 4:**
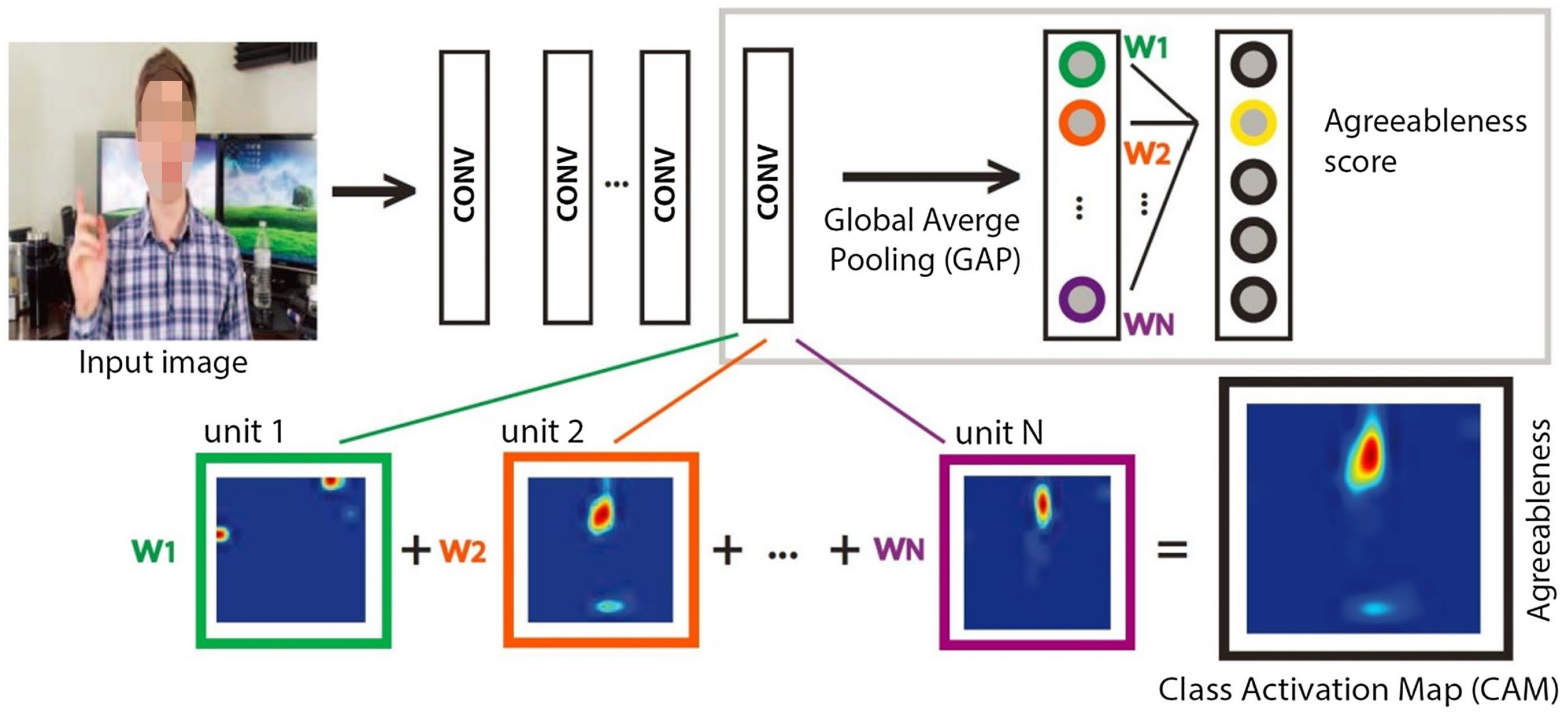
The used class activation maps (CAM) to interpret the CNN models in learning facial features (Ventura et al., 2017).

In consideration of the tremendous progress in the areas of deep learning, CNNs and LSTMs are widely for personality trait analysis from dynamic video sequences. Gürpınar et al. (2016) fine-tuned a pretrained VGG-19 network to extract deep facial and scene feature representations, as shown in Figure 3. Then, they were merged and fed into a kernel extreme learning machine (ELM) regressor for first impression estimation. Ventura et al. (2017) adopted an extension of Descriptor Aggregation Networks (DAN) to investigate why CNN models performed well in automatically predicting first impressions. They used class activation maps (CAM) for visualization and provided a possible interpretation on understanding why CNN models succeeded in learning discriminative facial features related to personality traits of users. Figure 4 shows the used CAM to interpret the CNN models in learning facial features. Experimental results indicate that: (1) face presents most of discriminative information for the inference of personality traits, (2) the internal representations of CNNs primarily focus on crucial facial regions including eyes, nose, and mouth, (3) some action units (AUs) provide a partial impact on the inference of facial traits. Beyan et al. (2019) aimed to perceive personality traits by means of using deep visual activity (VA)-based features derived only from key-dynamic images in videos. In order to determine key-dynamic images in videos, they employed three key steps: construction of multiple dynamic images, long-term VA learning with CNN + LSTM, and spatio-temporal saliency detection.

### Other Modality-Based Personality Trait Recognition

In addition to the above-mentioned audio and visual modality, there are other single modalities, such as text, and physiological signals, etc., which can be applied for personality trait recognition. Table 5 gives a brief summary of personality trait recognition based on text and physiological signals.

**Table 5:** A brief summary of text and physiological-based personality trait recognition.

Input type	Year	Authors	Feature descriptions
	2013	Bazelli et al.	Predicting the personality traits of Stack Overflow authors with LIWC
	2016	Golbeck et al.	The Receptiviti API providing personality score predictions with LIWC
	2017	Majumder et al.	A CNN with injection of the document-level Mairesse features
	2017	Hernandez et al.	RNNs and its variants, such as GRU, LSTM, and Bi-LSTM for text features
	2018	Xue et al.	A hierarchical deep neural network for learning deep semantic features
	2018	Sun et al.	A 2CLSTM integrating a Bi-LSTM with a CNN for feature extraction
	2020	Mehta et al.	Psycholinguistic features were combined with BERT embeddings
	2021	Ren et al.	A BERT for text feature extraction, followed by GRU, LSTM, and CNN
Physiological signals	2014	Wache et al.	The measurements of ECG, EEG, GSR
	2018	Subramanian et al.	The measurements of ECG, EEG, GSR and facial activity data
	2020	Taib et al.	Adopting eye-tracking and skin conductivity sensors for capturing their physiological responses

#### Text-Based Personality Trait Recognition

The text modality can effectively display traces of the user’s personality (Golbeck et al., 2011). One of the early-used features from text is the popular linguistic inquiry and word count (LIWC; Pennebaker et al., 2001), which is often used to extract lexical features. LIWC divides the words into a variety of psychologically buckets, such as function words (e.g., conjunctions and pronouns), affective words (e.g., amazing and cried), and so on. Then, the used frequency of different categories of words is counted in each bucket in purpose of predicting the personality traits of the writer. Bazelli et al. (2013) predicted the personality traits of Stack Overflow authors by means of analyzing the community’s questions and answers on the basis of LIWC. The recently developed Receptiviti API (Golbeck, 2016) is a popular tool using LIWC for personality trait prediction from text in psychology studies.

**Figure 5:**
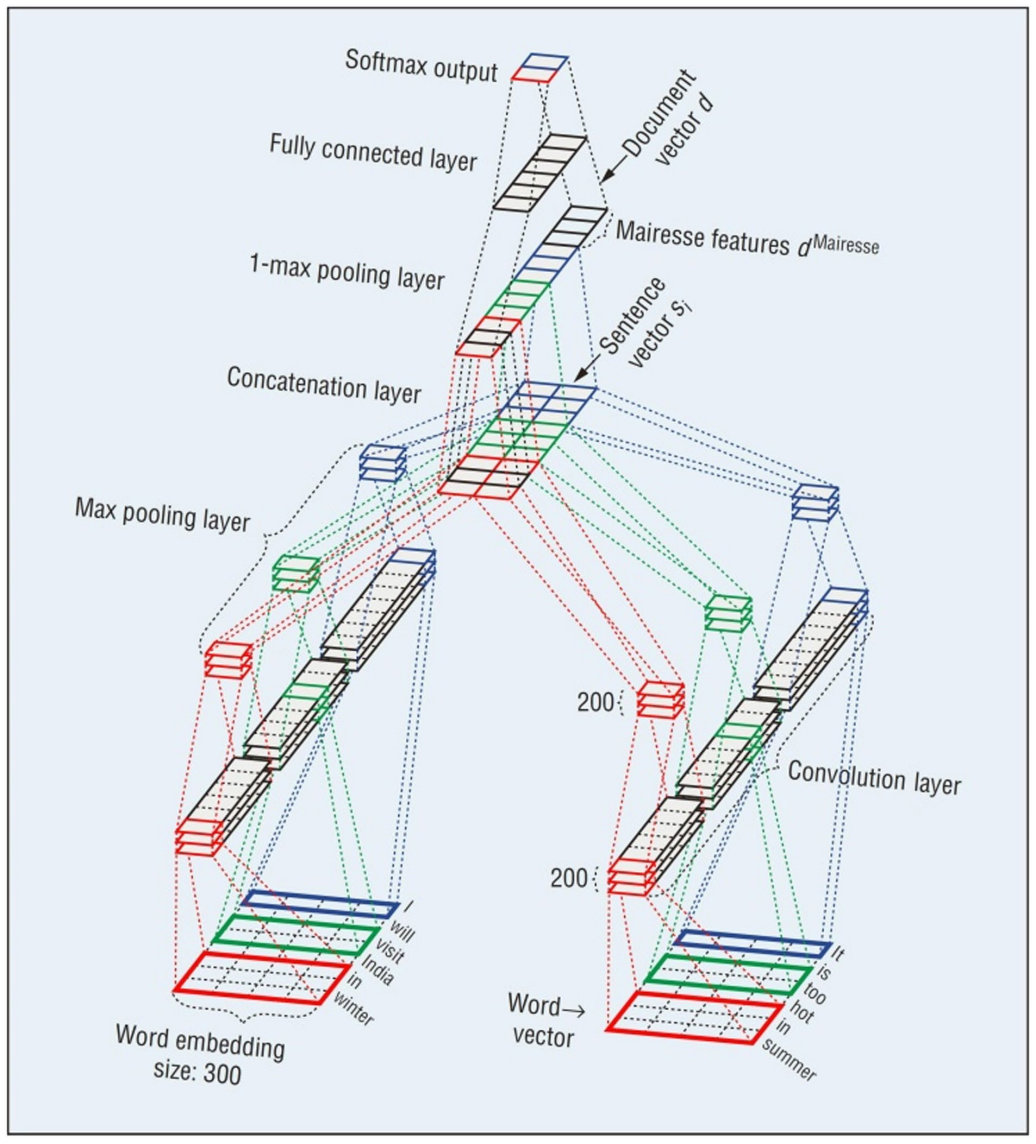
The flowchart of CNN-based document-level personality prediction from text (Majumder et al., 2017).

In recent years, several deep learning techniques have been employed for text-based personality trait recognition. Majumder et al. (2017) proposed a deep CNN method for document-level personality prediction from text, as depicted in Figure 5. The used CNN model consists of seven layers and aims to extract the monogram, bigram, and trigram features from text. Hernandez and Scott (2017) aimed at learning temporal dependencies among sentences by feeding the input text data into simple RNNs and its variants, such as GRU, LSTM, and Bi-LSTM. It was found that LSTM achieved better performance compared to RNN, GRU, and Bi-LSTM on MBTI personality trait recognition tasks. Xue et al. (2018) adopted a hierarchical deep neural network, including an attentive recurrent CNN structure and a variant of the inception structure, to learn deep semantic features from text posts of online social networks for the Big-five personality trait recognition. Sun et al. (2018) presented a model called 2CLSTM, integrating a Bi-LSTM with a CNN, for predicting user’s personality on the basis of structures of texts. Mehta et al. (2020a) proposed a deep learning-based model in which conventional psycholinguistic features were combined with language model embeddings like Bidirectional Encoder Representation From Transformers (BERT; Devlin et al., 2018) for personality trait prediction. Ren et al. (2021) presented a multilabel personality prediction model *via* deep learning, which integrated semantic and emotional features from social media texts. They conducted sentence-level extraction of both semantic and emotion features by means of a BERT model and a SentiNet5 (Vilares et al., 2018) dictionary model, respectively. Then, they fed these features into GRU, LSTM, and CNN for further feature extraction and classification. It was found that BERT+CNN performed best on MBTI and Big-Five personality trait classification tasks.

### Physiological Signal-Based Personality Trait Recognition

Since the user’s physiological responses to affective stimuli are highly correlated with personality traits, numerous works have tried to carry out physiological signal-based personality recognition. Wache (2014) investigated emotional states and personality traits on the basis of physiological responses to affective video clips. When watching 36 affective video clips, they utilized the measurements of Electrocardiogram (ECG), Galvanic Skin Response (GSR), Electroencephalogram (EEG) to characterize their Big-Five personality traits. Moreover, they also provided a multimodal database for implicit personality and affect classification by means of commercial physiological sensors (Subramanian et al., 2016). Taib et al. (2020) proposed a method of personality detection from physiological responses to affective image and video stimuli. They adopted eye-tracking and skin conductivity sensors for capturing their physiological responses.

## Multimodal Fusion for Personality Trait Recognition

For multimodal fusion on personality trait recognition tasks, there are generally three types: feature-level fusion, decision-level fusion, and model-level fusion (Zeng et al., 2008; Atrey et al., 2010).

Feature-level fusion aims to directly concatenate the extracted features from multimodal modalities, into one feature set. Therefore, feature-level fusion is also called early fusion (EF). As the simplest way of implementing feature integration, feature-level fusion has relatively low cost and complexity. Moreover, it considers the correlation between modalities. However, integrating different time scale and metric level of features from multimodal modalities will significantly increase the dimensionality of the concatenated feature vector, resulting in the difficulty of training models.

In decision-level fusion, each modality is firstly modeled independently, and then these obtained results from single-modality are combined to produce final results by using a certain number of decision fusion rules. Decision-level fusion is thus called late fusion (LF). The commonly used decision fusion rules include “Majority vote,” “Max,” “Sum,” “Min,” “Average,” “Product,” etc. (Sun et al., 2015). Since decision-level fusion considers different modalities as mutually independent, it can easily deal with asynchrony among modalities, resulting in the scalability with the number of modalities. Nevertheless, it fails to make use of the correlation between modalities at feature-level.

Model-level fusion aims to separately model each modality while taking into account the correlation between modalities. Therefore, it can consider the inter-correlation among different modalities and loose the demand of timing synchronization of these modalities.

Table 6 shows a brief summary of multimodal fusion for personality trait recognition. In the following, we present an analysis of these multimodal fusion methods from two aspects: bimodal and trimodal modalities for personality trait recognition.

**Table 6:** A brief summary of multimodal fusion for personality trait recognition.

Year	Authors	Modalities	Fusion methods	Feature descriptions
2016	Güçlütürk et al.	Audio, visual	Feature-level	An deep residual network for audio and visual feature extraction
2016, 2017	Zhang et al.	Audio, visual	Decision-level	A DBR method integrating audio and visual (scene and face) modality
2016	Gürpinar et al.	Audio, visual	Score-level	Fine-tuning a pretrained VGG model to derive facial emotion and ambient features. The INTERSPEECH-2009 for audio feature set
2016	Subramaniam et al.	Audio, visual	Feature-level	A volumetric (3D) convolution network for visual feature extraction. The statistics of zero-crossing rate, energy, MFCCs for audio features
2021	Curto et al.	Audio, visual	Model-level	The pretrained VGGish for audio feature extraction, and the pretrained R(2 + 1)D for video feature extraction
2016	Xianyu et al.	Text, visual	Model-level	A heterogeneity entropy (HE) neural network (HENN) consisting of HE-DBN, HE-AE and common DBN for common feature representations among text, image and behavior statistical modalities
2019	Principi et al.	Audio, visual	Model-level/Feature-level	A multimodal deep learning model (ResNet-50 for visual modality and 14-layer 1D CNN for audio modality) for feature extraction
2020	Li et al.	Audio, visual, text	Feature-level	A deep CR-Net to predict the multimodal Big-Five personality traits based on video, audio, and text cues
2017	Güçlütürk et al.	Audio, visual, text	Feature-level	A deep residual networks for audio-visual feature extraction. A bag-of-words and a skip-thought vector model for text feature extraction
2017, 2018	Gorbova et al.	Audio, visual, text	Decision-level	Acoustic LLD features (MFCCs, ZCR, speaking rate), facial action unit features, as well as negative and positive word scores
2018	Kampman et al.	Audio, visual, text	Decision-level/Model-level	An trimodal deep CNN method for audio, visual, text feature extraction
2020	Escalante et al.	Audio, visual, text	Feature-level	A bag-of-words model and a skip-thought vector model for text feature extraction, and the ResNet18 model for audio-visual feature extraction
2022	Suman et al.	Audio, visual, text	Feature-level/Decision-level	A MTCNN and ResNet for facial and ambient feature extraction, respectively. A VGGish model for audio feature extraction and an *n*-gram CNN model for text feature extraction

### Bimodal Modalities Based Personality Trait Recognition

For bimodal modalities based personality trait recognition, the widely used one is audio-visual modality. In order to effectively extract audio-visual feature representations of short video sequences, numerical studies have been conducted for audio-visual personality trait recognition.

Güçlütürk et al. (2016) developed an end-to-end audio-visual deep residual network for audio-visual apparent personality trait recognition. In detail, the audio data and visual data were firstly extracted from the video clip. Then, the whole audio data were fed into an audio deep residual network for feature learning. Note that the activities of the penultimate layer in the audio deep residual network were temporally pooled. Similarly, the whole visual data were fed into a visual deep residual network with a frame at a time. The activities of the penultimate layer in the visual deep residual network were spatiotemporally pooled. Finally, the pooled activities of the audio and visual stream were concatenated at feature-level as an input of a fully connected layer for personality trait prediction.

**Figure 6:**
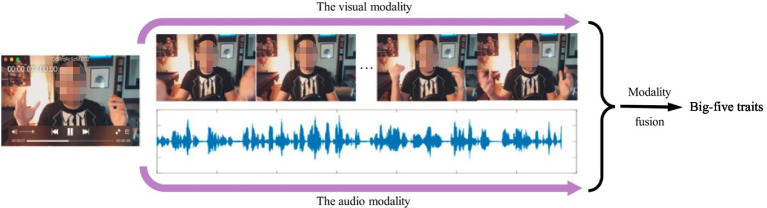
The flowchart of the proposed DBR method for audio-visual personality trait prediction (Wei et al., 2017).

Zhang et al., developed a deep bimodal regression (DBR) method so as to capture rich information from the audio and visual modality in videos (Zhang et al., 2016; Wei et al., 2017). Figure 6 shows the flowchart of the proposed DBR method audio-visual personality trait prediction. In particular, for visual feature extraction, they modified the traditional CNNs by means of discarding the fully connected layers. Additionally, they merged the average and max pooled features of the last convolutional layer into a whole feature vector, followed by the standard L2 normalization. For audio feature extraction, they extracted the logfbank features from the original audio utterances of videos. Then, they trained the linear regressor to produce the Big-Five trait values. To integrate the complementary cues from the audio-visual modality, they fused these predicted regression scores at decision-level.

Gürpinar et al. (2016) proposed a multimodal fusion method of audio and visual (scene and face) features for personality trait analysis. They fine-tuned a pretrained VGG model to derive facial emotion and ambient information from images. They also extracted local Gabor binary patterns from three orthogonal planes (LGBP-TOP) video descriptor as video features. The typical acoustic features, such as the INTERSPEECH-2009, 2010, 2012, and 2013 feature set in computational paralinguistics challenges, were employed. The kernel ELM was adopted for personality trait prediction on audio and visual (scene and face) modalities. Finally, a score-level method was leveraged to fuse the results of different modalities.

Subramaniam et al. (2016) employed two end-to-end deep learning models for audio-visual first impression analysis. They used a volumetric (3D) convolution network for visual feature extraction from face aligned images. For audio feature extraction, they obtained the statistics, such as mean and standard deviation of hand-crafted features like zero-crossing rate, energy, MFCCs, etc. Then, they concatenated the extracted audio and visual features at feature-level, followed by a multimodal LSTM network of temporal modeling for final personality trait prediction tasks.

Xianyu et al. (2016) proposed an unsupervised cross-modal feature learning method, called heterogeneity entropy (HE) neural network (HENN), for multimodal personality trait prediction. The proposed HENN consists of HE-DBN, HE-AE, and common DBN and is used to learn common feature representations among text, image, and behavior statistical modalities, and then map them into the user’s personality. The input of HENN is hand-crafted features. In particular, a bag of textual word (BoTW; Li et al., 2016) model was used to extract the text feature vector. Based on the extracted scale-invariant feature transform (SIFT; Cruz-Mota et al., 2012) features of each image, a bag of visual word model was used to produce visual image features. The time series information related to sharing numbers and comment numbers in both text and image modalities were employed to compute behavior statistical parameters. These hand-crafted features were individually fed into three HE-DBNs for initial feature learning, and then HE-AE and common DBN were separately adopted to fuse these features produced with HE-DBNs at model-level for final Big-Five personality prediction.

Principi et al. (2019) developed a multimodal deep learning model combining the raw visual with audio streams to conduct the Big-Five personality trait prediction. For each video sample, different task-specific deep models, related to individual factor, such as facial expressions, attractiveness, age, gender, and ethnicity, were leveraged to estimate per-frame attribute. Then, these estimated results were concatenated at feature-level to produce a video-level attribute prediction by spatio-temporal aggregation methods. For visual feature extraction, they adopted a ResNet-50 network pretrained on the ImageNet data to produce high-level feature representations on each video frame. For audio feature extraction, a 14-layer 1D CNN like the ResNet-18 was used. They fused these modalities in two steps. First, they employed a FC layer for model-level fusion to learn the joint feature representations of the concatenated video-level attribute predictions. This model-level fusion step was also used to reduce the dimensionality of the concatenated video-level attribute predictions. Second, they combined such learned joint video-level attribute predictions with the extracted audio and visual features at feature-level, to perform final the Big-Five personality trait prediction.

Curto et al. (2021) developed the Dyadformer for modeling individual and interpersonal audio-visual features in dyadic interactions for personality trait prediction. The Dyadformer was a multimodal multisubject Transformer framework consisting of a set of attention encoder modules (self, cross-modal, and cross-subject) with Transformer layers. They employed the pretrained VGGish (Hershey et al., 2017) model to produce a 128-dimensional embedding for each audio chunk. They leveraged the pretrained R(2 + 1)D (Tran et al., 2018) model to generate a 512-dimensional embedding for each video chunk. They used cross-modal and cross-subject attentions for multimodal Transformer fusion in model-level.

### Trimodal Modalities Based Personality Trait Recognition

Li et al. (2020b) presented a deep classification–regression network (CR-Net) to predict the multimodal Big-Five personality traits based on video, audio, and text cues and further applied to the job interview recommendation. For the visual input, they extracted the global scene cues and local face cues by using the ResNet-34 network. Considering audio-text inner correlations, they concatenated the extracted acoustic LLD and text-based skip-thought vectors at feature-level as inputs of the ResNet-34 network for audio-text feature learning. Finally, they merged all extracted features from visual, audio, and text modalities at feature-level and fed them into the CR-Net network to analyze the multimodal Big-Five personality traits.

Güçlütürk et al. (2017) presented a method of multimodal first impression analysis integrating audio, visual, and text (language) modalities, based on deep residual networks. They adopted two similar 17-layer deep residual networks for extracting audio-visual features. The used 17-layer deep residual networks consist of one convolutional layer and eight residual blocks of two convolutional layers. The pooled activities of audio-visual networks were concatenated as an input of a fully connected layer so as to learn the joint audio-visual feature representations. For text feature extraction, they utilized two language models, including a bag-of-words model and a skip-thought vector model, to produce the annotations as a function of the language data. Both of the language models contain an embedding layer, followed by a linear layer. Finally, they combined the extracted features from audio, visual, and text at feature-level for the multimodal Big-five personality trait analysis and job interview recommendation.

**Figure 7:**
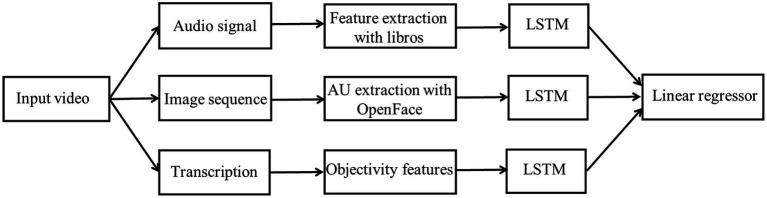
The flowchart of Integrating audio, vision and language for first-Impression personality analysis (Gorbova et al., 2018).

Gorbova et al. (2017, 2018) provided an automatic personality screening method on the basis of visual, audio, and text (lexical) cues from short video clips for predicting the Big-five personality traits. The extracted hand-crafted features contained acoustic LLD features (MFCCs, ZCR, speaking rate, etc.), facial action unit features, as well as negative and positive word scores. This system adopted the weighted average strategy to fuse the final obtained results from three modalities at decision-level. Figure 7 shows the flowchart of integrating audio, vision, and language for first impression personality analysis (Gorbova et al., 2018). In Figure 7, after extracted audio, visual, and lexical features from input video, three separate LSTM cells were used for modeling long dependency. Then, the hidden features in LSTMs were processed by a linear regressor. Finally, the obtained results were fed to an output layer for the Big-five personality trait analysis.

Kampman et al. (2018) presented an end-to-end trimodal deep learning architecture for predicting the Big-Five personality traits by means of integrating audio, visual, and text modalities. For audio channel, the raw audio waveform and its energy components with squared amplitude were fed into a CNN network with four convolutional layers and a global average pooling layer for audio feature extraction. For visual channel, based on a random frame image of a video, they fine-tuned the pretrained VGG-16 model for video feature extraction. For text channel, they adopted “Word2vec” word embedding from transcriptions as an input of a CNN network for text feature extraction. In this text CNN network, three different convolutional windows corresponding to three, four, and five words over the sentence were used. Finally, they fused audio, visual, and text modalities at both decision-level and model-level. For decision-level fusion, a voting scheme was used. For model-level fusion, by means of concatenating the output of FC layers of each CNN, they added another two FC layers on top to learn shared feature representations of input trimodal data.

Escalante et al. explored the explainability in first impressions analysis from video sequences at the first time. They provided a baseline method of integrating audio, visual, and text (audio transcripts) information (Escalante et al., 2020). They used a variant of original 18-layer deep residual networks (ResNet-18) for audio and visual feature extraction, respectively. The feature-level fusion was adopted after the global average pooling layers of the audio-visual ResNet-18 models *via* concatenation of their obtained latent features. For text modality, two language models, such as a skip-thought vector model and a bag-of-words model, were employed for text feature extraction. Finally, a concatenation of audio, visual, text-based latent features was implemented at feature-level for multimodal first-impression analysis.

Suman et al. (2022) developed a deep learning-based multimodal personality prediction system integrating audio, visual, and text modalities. They extracted facial and ambient features from the visual modality by using Multi-task Cascaded Convolutional Neural Networks (MTCNN; Jiang et al., 2018) and ResNet, individually. They extracted the audio features by using a VGGish (Hershey et al., 2017) model, and the text features by using an *n*-gram CNN model, respectively. These extracted audio, visual, and text features were fed into a fully connected layer followed by a sigmoid function for the final personality trait prediction. It was concluded that the concatenation of audio, visual, and text features in feature-level fusion showed comparable performance with the averaging method in decision-level fusion.

## Challenges and Opportunities

To date, although there are a number of literature related to multimodal personality trait prediction, showing its certain advance, a few challenges still exist in this area. In the following, we discuss these challenges and opportunities, and point out potential research directions in future.

### Personality Trait Recognition Data Sets

Although researchers have developed a variety of relevant data sets for personality trait recognition, as shown in Table 1, these data sets are relatively small. To date, the most popular multimodal data sets, such as the ChaLearn First Impression V1 (Ponce-López et al., 2016), and its enhanced version V2 (Escalante et al., 2017), consist of 10,000 short video clips. Such data sets are definitely smaller, compared with the well-known ImageNet data set with a total of 14 million images used for training deep models. Considering that automatic personality trait recognition is a data-driven task associated with a deep neural network, a large amount of training data is required for training sufficiently deep models. Therefore, one major challenge for deep multimodal personality trait recognition is the lack of a large amount of training data on the basis of both quantity and quality.

In addition, owing to the difference of data collecting and annotating environment, data bias and inconsistent annotations usually exist among these different data sets. Most researchers conventionally verify the performance of their proposed methods within a specific data set, resulting in promising results. Such trained models based on intra-data set protocols commonly lack generalizability on unseen test data. Therefore, it is interesting to investigate the performance of multimodal personality trait recognition methods in cross-data set environment. To address this issue, deep domain adaption methods (Wang et al., 2020; Kurmi et al., 2021; Shao and Zhong, 2021) may be an alternative. Note that the display of personality traits and the traits themself can be considered as context-dependent. This will also give a considerable challenge for the training models on personality trait recognition tasks.

### Integrating More Modalities

For multimodal personality trait recognition, bimodal modalities like audio-visual, or trimodal modalities like audio, visual, and text, are usually employed. Note that the user’s physiological responses to affective stimuli are highly correlated with personality traits. However, few researchers explore the performance of integrating physiological signals with other modalities for multimodal personality trait recognition. This is because so far these are few multimodal personality trait recognition data sets, which incorporate physiological signals with other modalities. Hence, one may challenge is how to combine physiological signals and other modalities, such as audio, visual, and text clues, based on the corresponding developed multimodal data sets.

Besides, other behavior signals, such as head and body pose information, which is related to personality trait clues (Alameda-Pineda et al., 2015), may present complementary information to further enhance the robustness of multimodal personality trait recognition. It is thus a promising research direction to integrate head and body clues with existing modalities, such as audio, visual, and text clues for multimodal personality trait recognition.

### Limitations of Deep Learning Techniques

So far, a variety of representative deep leaning methods have been successfully applied to learn high-level feature representations for automatic personality trait recognition. Moreover, these deep learning methods usually beat other methods adopting hand-crafted features. Nevertheless, these used deep learning techniques have a tremendous amount of network parameters, resulting in its large computation complexity. In this case, for real-time application sceneries it is often difficult to implement fast automatic personality trait prediction with these complicated deep models. To alleviate this issue, a deep model compression (Liang et al., 2021a; Tartaglione et al., 2021) may present a possible solution.

Although deep learning has become a state-of-the-art technique in term of the performance measure on various feature learning tasks, the black box problem still exists. In particular, it is unknown that what exactly are various internal representations learned by multiple hidden layers of a deep model. Owing to its multilayer non-linear structure, deep learning techniques are usually criticized to be non-transparent, and their prediction results are often not traceable by human beings. To alleviate this problem, directly visualizing the learned features has become the widely used way of understanding deep models (Escalante et al., 2020). Nevertheless, such visualizing way does not really present the related theories to explain what exactly this algorithm is doing. Therefore, it is an important research direction to explore the explainability and interpretability of deep learning techniques (Tjoa and Guan, 2020; Krichmar et al., 2021; Liang et al., 2021b; Yan et al., 2021) from a theoretical perspective for automatic personality trait recognition.

### Investigating Other Trait Theories

It is noted that most researchers focus on personality trait analysis *via* the Big-Five personality model. This is because almost all of the current data sets were developed based on the Big-Five personality measures, as shown in Table 1. However, very few literature concentrate on other personality measures, such as the MBTI, PEN, and 16PF, due to the lacking data resources. In particular, the MBTI personality measure, as the most popular administered personality test throughout the world, shows more difficulty in prediction than the Big-Five model (Furnham and Differences, 1996; Furnham, 2020). Therefore, it is an open issue to investigate the effect of other trait theories on personality trait prediction on the basis of correspondingly constructed data sets.

## Conclusion

Due to the strong feature learning ability of deep learning, multiple recent works using deep learning have been developed for personality trait recognition associated with promising performance. This paper attempts to provide a comprehensive survey of existing personality trait recognition methods with specific focus on hand-crafted and deep learning-based feature extraction. These methods systematically review the topic from the single modality and multiple modalities. We also highlight numerous issues for future challenges and opportunities. Apparently, personality trait recognition is a very broad and multidisciplinary research issue. This survey only focuses on reviewing existing personality trait recognition methods from a computational perspective and does not take psychological studies into account on personality trait recognition.

In future, it is interesting to explore the application of personality trait recognition techniques to personality-aware recommendation systems (Dhelim et al., 2021). In addition, since personality traits are usually strongly connected with emotions, it is an important direction to investigate a CNN-based multitask learning framework for emotion and personality detection (Li et al., 2021).

## Author Contributions

XZ contributed to the writing and drafted this article. ZT contributed to the collection and analysis of existing literature. SZ contributed to the conception and design of this work and revised this article. All authors contributed to the article and approved the submitted version.

## Funding

This work was supported by Zhejiang Provincial National Science Foundation of China and National Science Foundation of China (NSFC) under Grant Nos. LZ20F020002, LQ21F020002, and 61976149.

## Conflict of Interest

The authors declare that the research was conducted in the absence of any commercial or financial relationships that could be construed as a potential conflict of interest.

## Publisher’s Note

All claims expressed in this article are solely those of the authors and do not necessarily represent those of their affiliated organizations, or those of the publisher, the editors and the reviewers. Any product that may be evaluated in this article, or claim that may be made by its manufacturer, is not guaranteed or endorsed by the publisher.
